# Do urinary and double incontinence predict changes in living arrangements and mobility in older women after hip fracture? – a 1-year prospective cohort study

**DOI:** 10.1186/s12877-023-04637-z

**Published:** 2024-01-26

**Authors:** Aino T. Hellman-Bronstein, Tiina H. Luukkaala, Seija S. Ala-Nissilä, Maria S. Nuotio

**Affiliations:** 1 Department of Geriatric Medicine, Turku University Hospital and University of Turku, Turku, Finland; 2 Research, Development and Innovation Center, Tampere University Hospital, Tampere, Finland; 3 Health Sciences, Faculty of Social Sciences, Tampere University, Tampere, Finland; 4 Department of Obstetrics and Gynecology, Turku University Hospital and University of Turku, Turku, Finland; 5 Department of Geriatric Medicine, Seinäjoki Central Hospital, Seinäjoki, Finland

**Keywords:** Urinary incontinence, Double incontinence, Institutionalization, Hip fracture, Mobility

## Abstract

**Background:**

Continence problems are known to be associated with disability in older adults. Costs of disability and resulting need for more supported living arrangements are high after a hip fracture. The aim was to examine pre-fracture urinary incontinence (UI) and double incontinence (DI, concurrent UI and fecal incontinence) as predictors of changes in mobility and living arrangements in older female hip fracture patients in a 1-year follow-up.

**Methods:**

Study population comprised 1,675 female patients aged ≥ 65 (mean age 82.7 ± 6.8) sustaining their first hip fracture between 2007–2019. Data on self-reported pre-fracture continence status was collected. The outcomes were declined vs. same or improved mobility level and need for more assisted vs same or less assisted living arrangements 1-year post-fracture. Separate cohorts of 1,226 and 1,055 women were generated for the mobility and living arrangements outcomes, respectively. Age- and multivariable-adjusted logistic regression models were used to determine the associations of UI, DI, and other baseline characteristics with the outcomes.

**Results:**

Of the patients, 39% had declined mobility or more assisted living arrangements at 1-year follow-up. Adjusting for age, both pre-fracture UI and DI were associated with changes in mobility and living arrangements. In the multivariable analysis, UI (OR 1.88, 95% CI 1.41–2.51) and DI (1.99, 95% CI 1.21–3.27) were associated with decline in mobility level while only DI (OR 2.40, 95% CI 1.22–4.75) remained associated with the need for more assisted living arrangements.

**Conclusions:**

Both pre-fracture UI and DI in older women are risk factors for declining mobility level, but only DI for more supported living arrangements 1-year post-hip fracture. UI likely develops earlier in life and might not necessarily be strongly associated with the onset or increasing disability in later years. DI may indicate more marked vulnerability and burden to patients as well as to formal and informal caregivers.

## Background

Urinary incontinence (UI), defined as any reported involuntary loss of urine [1], is more common among women than among men and its prevalence increases with advancing age. Nearly 40% of women older than 60 years and 80% of women residing in long-term care have UI [2, 3]. UI has long been recognized as one of the most common geriatric syndromes and it is associated with multiple poor outcomes. As opposed to UI as an urogynecologic condition in younger women, factors leading to the development of UI in older women are complex and often related to multifactorial health conditions and functional limitations i.e., factors outside the urinary tract [2, 4]. In addition, these factors are not exclusive to incontinence but also to other geriatric syndromes, such as dizziness and falls [5]. In an observational study of nearly 30,000 women aged 65 and over, 80% had at least one geriatric syndrome, and over 10% had at least three, UI being the most common. Women with multiple geriatric syndromes had a higher risk of disability in the 3-year follow-up [6]. It is therefore not surprising that older individuals with UI are from 1.5 to 2.3 times more likely to fall than are individuals without urinary symptoms [7]. Hip fracture, one of the most devastating consequences of a fall, is a common injury among older women with well-established consequences of increased mortality, morbidity, and costs [8, 9]. Loss of independence and mobility and the resulting need for institutionalization amount to almost one-third of the total costs of the first year following a hip fracture [10]. Advancing age and cognitive impairment have been most consistently reported predictors of institutionalization after a hip fracture [11, 12].

Double incontinence (DI), defined as concurrent UI and fecal incontinence (FI) [13], is also more common in women and associated with advancing age. It is a severe manifestation of pelvic floor dysfunction and associated with functional, physical, and cognitive deficits. According to earlier studies, prevalence of DI has ranged between 2.5 and 14.5% in community-dwelling older individuals [14–16] and 33–65% in patients residing in long term care [17, 18].

Few studies have examined the role of incontinence as a predictor of institutionalization, and this association has mostly been demonstrated for men, not women [19–21]. Importantly, it has been estimated that UI adds an annual 6 billion dollars to the total costs of long-term care admissions in the US [22]. Although several articles state that DI is believed to be a frequent cause for referrals to long-term care [15, 16, 23], we found no actual studies examining the association of DI with institutionalization.

We have previously established UI and DI to be very common among older women with hip fracture and especially DI to be related to disability [24, 25]. We have demonstrated that older women with non-independent mobility had a higher risk of developing incident UI and DI compared to independent mobilizers during 1-year follow-up after a hip-fracture [25]. Conversely, physical activity has been shown to protect older women against incident incontinence [26]. However, we found only few studies examining an inverse relationship of UI predicting decline in mobility, and none on DI.

Since older women frequently suffer from both incontinence and hip fractures, we found it important to evaluate the significance of pre-fracture UI and DI as predictors of need for more assisted living arrangements and decline in mobility level 1-year post-fracture. Our aim was to further elucidate the importance of managing incontinence during the rehabilitation process after a hip fracture.

## Methods

### Study population

We have described the study population in detail in our previous publications [24, 25]. In brief, the data consisted of 1,675 consecutive female patients aged ≥ 65 sustaining hip fracture between September 2007 and January 2019 in the Hospital District of Southern Ostrobothnia, with a population of approximately 200,000 inhabitants. Patients were managed in Seinäjoki Central Hospital, which is the only hospital providing acute surgical care in the area. Only women with their first hip fracture during the follow-up period were included. Patients with pathologic and periprosthetic fractures were excluded. Patients were transferred to primary care hospitals of seven health care districts for rehabilitation. The median length of stay in the acute hospital providing trauma care was five days (interquartile range [IQR] of 2–8 days).

**Fig. 1:**
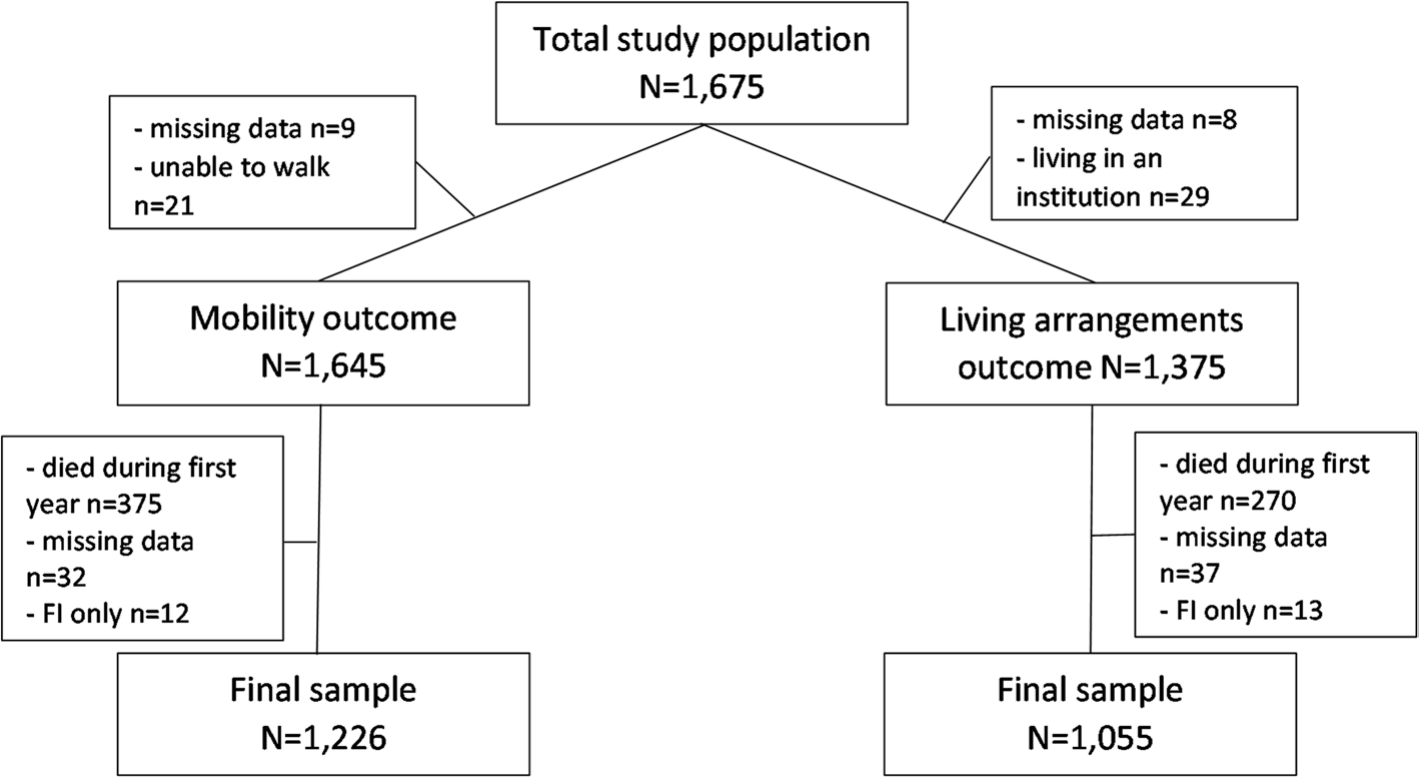
Flow chart of the study population

Two separate study cohorts were generated for the mobility and living arrangements outcomes. For the mobility outcome, patients unable to ambulate at the time of the fracture were excluded from the final sample. Similarly for the living arrangements outcome, patients already living in an institution at the time of the fracture were excluded. Approximately 20% of the patients died during the first year after the fracture and follow-up data were missing in small proportion of the women. Finally, as in our previous works [24, 25], due to very small numbers, the few patients with FI only were excluded from the final samples, leaving 1,226 women eligible for analysis on changes in living arrangements and 1,055 women for analysis on changes in mobility at 1-year post-fracture, respectively. A flow chart of the study population is presented in Fig. 1.

### Data collection and variables

As described in our previous reports [24, 25], observational prospective data were collected at the time of the fracture during hospitalization and 1-year post-fracture by a geriatric nurse by face-to-face and telephone interviews with patients or their representatives, and from the patient medical records. In cases where the participants were unable to answer the questions by themselves, proxy respondents such as the next of kin were used. The study was approved by the Ethics Committee of the Hospital District of Southern Ostrobothnia. All the participants or their representatives gave informed consent.

We have previously studied pre- and perioperative predictors of changes in living arrangements and mobility in the whole cohort of older hip fracture patients including both men and women, and many of these predictor variables were used as covariates in the present study [27]. The variables used in the present study are described in greater detail elsewhere [24, 25, 27]. Given the potentially cognitively impaired respondents, simple survey-like questions were used instead of structured questionnaires to define UI and DI. UI was defined as any reported involuntary loss of urine (yes or no) and FI as any reported involuntary loss of feces respectively. Patients with both UI and FI were deemed to have DI. To observe changes over time, the pre-fracture and 1-year post-fracture living arrangements and mobility level were elicited in a similar way, as also described in our above mentioned earlier report [27]. As stated in that report, living arrangements were categorized as 1) living independently in own home, 2) living in own home with the assistance of organized home care or in an assisted living accommodation, and 3) living in an institution. Living in an institution was defined as residing in a primary care hospital or a long-term care facility (LTCF) providing 24-h care. Data on mobility level was also categorized based on simple survey-like questions and categorized as 1) being able to ambulate independently outdoors, 2) being able to ambulate independently indoors, outdoors with help, 3) being able to ambulate only indoors with help, 4) unable to ambulate. A modification of the original dataset of the British National Hip Fracture Database was applied to categorize the mobility level in our study [28]. As in our previous reports on Seinäjoki hip fracture data, the study outcomes were dichotomized into more assisted vs same or less assisted living arrangements and declined vs same or improved mobility level, respectively. This was done by categorizing the changes from aforementioned living arrangements and mobility levels to another between the time of fracture and one year of follow up [27].

For adjustment purposes in the analyses, data on age, number of prescribed medications, diagnosis of cognitive disorder (yes or no), fracture type, ASA score, the MNA-SF to describe the nutritional status of the respondents and removal or non-removal of urinary catheter (UC) before discharge from the central hospital were used. The diagnosis of cognitive disorder—as made by a geriatrician or a neurologist according to our national guidelines [29]—was ascertained from the patient medical records. Mild cognitive impairment was not included among the diagnoses.

The preoperative American Society of Anesthesiologists (ASA) risk scores were used to assess general health at baseline. There are five classes: (1) healthy person, (2) mild systemic disease, (3) severe systemic disease, (4) severe systemic disease that is a constant threat of life, and (5) a moribund person who is not expected to survive without surgery [30]. Nutritional status was assessed using the Mini Nutritional Assessment Short Form (MNA-SF) [31] during hospitalization. For the purposes of this study, poor nutrition was defined as being at risk for malnutrition or being malnourished according to the MNA-SF. Categorization of the baseline variables is shown in Tables 1 and 2. All of the abovementioned covariates have been described in greater details in our earlier reports [24, 25, 27].

**Table 1:** Age- and multivariable adjusted associations of baseline variables with changes in living arrangements (more assisted vs. same/less assisted) (*N*=1,055) after 1 year of follow-up

		**Same/less assisted** ***n*=647**		**More assisted** ***n*=408**		**Age-adjusted**		**Multivariable-adjusted**	
	N	n	(%)	n	(%)	OR	(95% CI)	OR	(95% CI)
**Age**									
65-79	325	241	(37)	84	(21)	1.00		1.00	
80-89	561	329	(51)	232	(57)	**2.02**	**(1.50-2.73)**	**2.00**	**(1.42-2.82)**
≥ 90	169	77	(12)	92	(23)	**3.43**	**(2.32-5.07)**	**4.30**	**(2.65-6.98)**
Fracture type*									
Intracapsular	658	403	(62)	255	(63)	1.00		1.00	
Extracapsular	394	243	(38)	151	(37)	0.29	(0.66-1.13)	0.91	(0.68-1.22)
**ASA**									
1-2	180	135	(21)	45	(11)	1.00		1.00	
3-5	860	501	(77)	359	(88)	**1.74**	**(1.19-2.53)**	1.67	(1.08-2.57)
Not known	15	11	(2)	4	(1)	1.00	(0.30-3.23)	0.87	(0.23-3.36)
**Number of regularly taken medications**									
< 4	230	147	(23)	83	(20)	1.00		1.00	
4-10	656	403	(62)	253	(62)	1.03	(0.75-1.42)	0.92	(0.64-1.34)
> 10	169	97	(15)	72	(18)	1.22	(0.80-1.84)	1.12	(0.68-1.84)
Diagnosis of cognitive disorder*									
No	838	559	(86)	279	(68)	1.00		1.00	
Yes	215	86	(13)	129	(32)	**2.83**	**(2.07-3.87)**	**2.87**	**(1.95-4.22)**
Mobility*									
Independently outdoors	699	498	(77)	201	(49)	1.00		1.00	
Independently indoors, outdoors with help	327	141	(22)	186	(46)	**2.78**	**(2.1-3.69)**	**3.61**	**(2.47-5.27)**
Indoors, only with help	21	6	(1)	15	(4)	**5.84**	**(2.21-15.4)**	**4.81**	**(1.60-14.4)**
Unable to ambulate	5	1	(0)	4	(1)	**10.2**	**(1.1-93.4)**	**17.0**	**(1.53-189)**
Living arrangements*									
Home without services	551	323	(50)	228	(56)	1.00		1.00	
Home with organized home care/assisted living accommodation	504	324	(50)	180	(44)	**0.57**	**(0.43-0.74)**	**0.16**	**(0.10-0.23)**
**MNA-SF before hip fracture**									
Normal (12-14)	490	333	(52)	157	(39)	1.00		1.00	
Poor nutrition (< 12)	315	162	(25)	153	(38)	**1.87**	**(1.39-2.51)**	1.41	(0.99-1.99)
Not known	250	152	(24)	98	(24)	**1.38**	**(1.00-1.91)**	1.01	(0.67-1.53)
**Removal of urinary catheter**									
During hospital stay	607	404	(62)	203	(50)	1.00		1.00	
Later	439	236	(37)	203	(50)	**1.61**	**(1.24-2.09)**	**1.67**	**(1.22-2.30)**
**Continence before fracture**									
Continent	497	337	(52)	160	(39)	1.00		1.00	
Urinary incontinent	411	242	(37)	169	(41)	**1.33**	**(1.01-1.76)**	1.26	(0.92-1.73)
Double incontinent	60	21	(3)	39	(10)	**3.77**	**(2.12-6.70)**	**2.40**	**(1.22-4.75)**
Not known	87	47	(7)	40	(10)	**1.72**	**(1.08-2.76)**	1.60	(0.96-2.90)

*ASA* American Society of Anesthesiologists -risk score, *MNA-SF* Mini Nutritional Assessment Short Form

*Small numbers of missing values (*n*<10) were not shown in table. Statistically significant results (*p*<0.05) were bolded

**Table 2:** Age- and multivariable adjusted associations of baseline variables with changes in mobility (declined vs. same/better) (*N* = 1,226) after 1 year of follow-up

		**Same/better *n* = 749**		**Declined *n* = 477**		**Age-adjusted**		**Multivariable-adjusted**	
	N	n	(%)	n	(%)	OR	(95% CI)	OR	(95% CI)
**Age**									
65–79	353	251	(34)	102	(21)	1.00		1.00	
80–89	662	399	(53)	263	(55)	**1.62**	**(1.23–2.14)**	1.23	(0.91–1.68)
≥ 90	211	99	(13)	112	(24)	**2.78**	**(1.95–3.97)**	**2.17**	**(1.45–3.24)**
Fracture type^*^									
Intracapsular	751	488	(65)	263	(55)	1.00		1.00	
Extracapsular	472	259	(35)	213	(45)	**1.40**	**(1.10–1.78)**	1.28	(0.99–1.66)
**ASA**									
1–2	186	140	(19)	46	(10)	1.00		1.00	
3–5	1021	597	(80)	424	(89)	**1.82**	**(1.27–2.62)**	1.39	(0.93–2.09)
Not known	19	12	(2)	7	(2)	1.56	(0.57–4.25)	1.37	(0.45–4.17)
**Number of regularly taken medications**									
< 4	239	162	(22)	77	(16)	1.00		1.00	
4–10	777	469	(63)	308	(65)	1.29	(0.95–1.77)	0.95	(0.67–1.35)
> 10	210	118	(16)	92	(19)	**1.51**	**(1.02–2.23)**	0.97	(0.62–1.51)
Diagnosis of cognitive disorder^*^									
No	889	594	(79)	295	(62)	1.00		1.00	
Yes	335	153	(20)	182	(38)	**2.30**	**(1.77–2.99)**	**2.00**	**(1.45–2.77)**
Mobility^*^									
Independently outdoors	714	455	(61)	259	(54)	1.00		1.00	
Independently indoors, outdoors with help	454	260	(35)	194	(41)	1.09	(0.85–1.40)	**0.40**	**(0.29–0.57)**
Indoors, only with help	58	34	(5)	24	(5)	1.08	(0.62–1.88)	**0.24**	**(0.12–0.47)**
Living arrangements^*^									
Home without services	552	400	(53)	152	(32)	1.00		1.00	
Home with organized home care/assisted living accommodation	500	271	(36)	229	(48)	**1.95**	**(1.49–2.54)**	**1.77**	**(1.29–2.43)**
Institution	171	75	(10)	96	(20)	**2.93**	**(2.04–4.22)**	**2.56**	**(1.58–4.15)**
**MNA-SF before hip fracture**									
Normal (12–14)	516	338	(45)	178	(37)	1.00		1.00	
Poor nutrition (< 12)	419	223	(30)	196	(41)	**1.55**	**(1.19–2.03)**	1.29	(0.95–1.76)
Not known	291	188	(25)	103	(22)	1.03	(0.76–1.40)	0.86	(0.59–1.24)
**Removal of urinary catheter**									
During hospital stay	691	470	(63)	221	(46)	1.00		1.00	
Later	526	272	(36)	254	(53)	**1.95**	**(1.54–2.48)**	**2.17**	**(1.64–2.86)**
**Continence before fracture**									
Continent	524	374	(50)	150	(31)	1.00		1.00	
Urinary incontinent	497	260	(35)	237	(50)	**2.11**	**(1.63–2.75)**	**1.88**	**(1.41–2.51)**
Double incontinent	109	51	(7)	58	(12)	**2.63**	**(1.72–4.03)**	**1.99**	**(1.21–3.27)**
Not known	96	64	(9)	32	(7)	1.19	(0.75–1.91)	0.98	(0.59–1.64)

*ASA* American Society of Anesthesiologists -risk score, *MNA-SF* Mini Nutritional Assessment Short Form

^*^Small numbers of missing values (n < 10) were not shown in table. Statistically significant results (*p* < 0.05) were bolded

### Statistical analyses

Age- and multivariable-adjusted logistic regression analyses with odds ratios (OR) and 95% confidence intervals (CI) were conducted to evaluate the association of pre-fracture UI and DI and other baseline variables with the outcome variables of changes in mobility and living arrangements 1-year post-fracture. IBM SPSS Statistics version 25.0 for Windows software (SPSS Inc. Chicago, Illinois) was used for statistical analyses. All tests were two-sided, and p values < 0.05 were considered statistically significant.

## Results

The mean age of the patients was 82.7 ± 6.8. Mortality rate in the mobility outcome cohort was 23%, and 20% in the living arrangements outcome cohort (Fig. 1). Out of the 1,055 women, 497 (47%) were continent, 411 (39%) had UI, and 60 (6%) had DI pre-fracture. Of these women, 647 (61%) required the same or less assisted living arrangements one-year post-fracture compared to the time before the fracture, and 408 (39%) required more assistance (Table 1). Out of the 1,226 women of the mobility outcome, 524 (43%) were continent, 497 (41%) had UI, and 109 (9%) had DI pre-fracture. Of these women, 749 (61%) had the same or better mobility-level at 1-year post-fracture, and 477 (39%) worse mobility, respectively (Table 2). In both groups, only small proportion of the patients had improved.

**Fig. 2:**
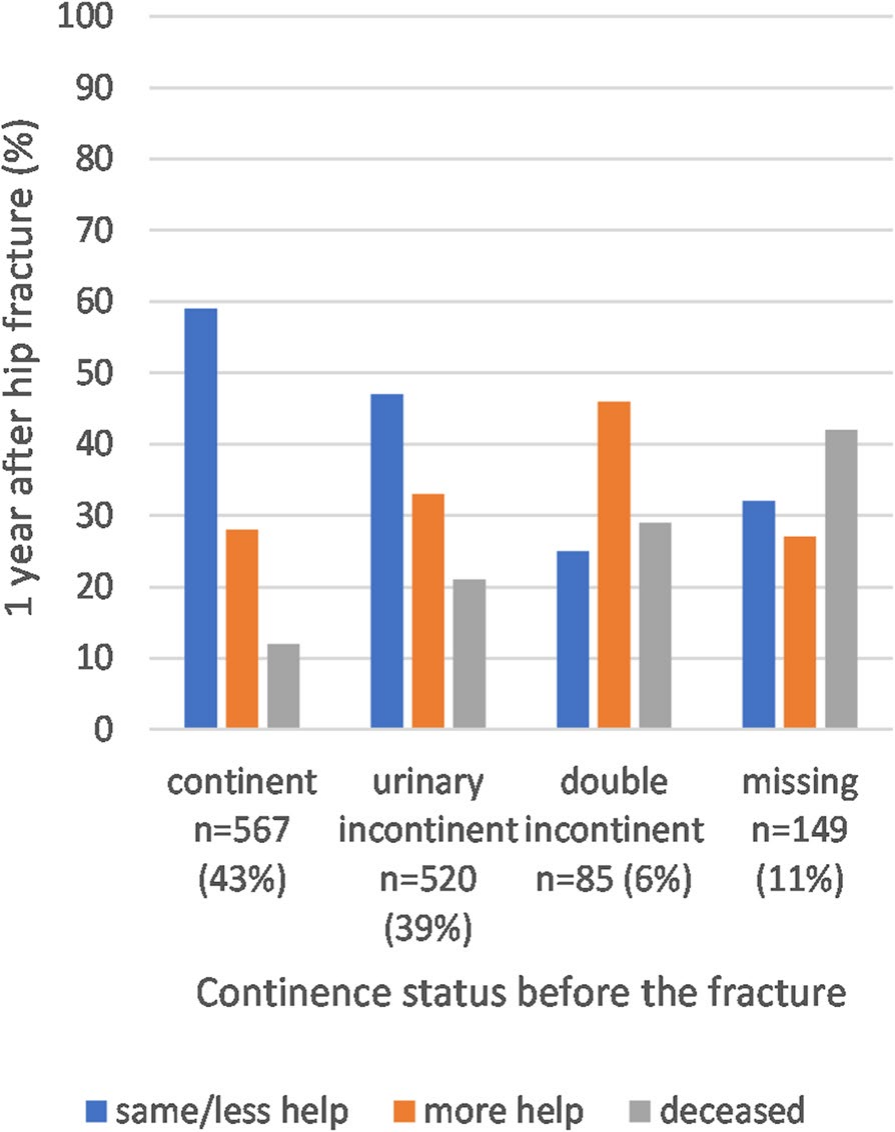
Changes in living arrangements after 1 year of follow-up according to pre-fracture continence status (*n* = 1321, 79% of the study population)

**Fig. 3:**
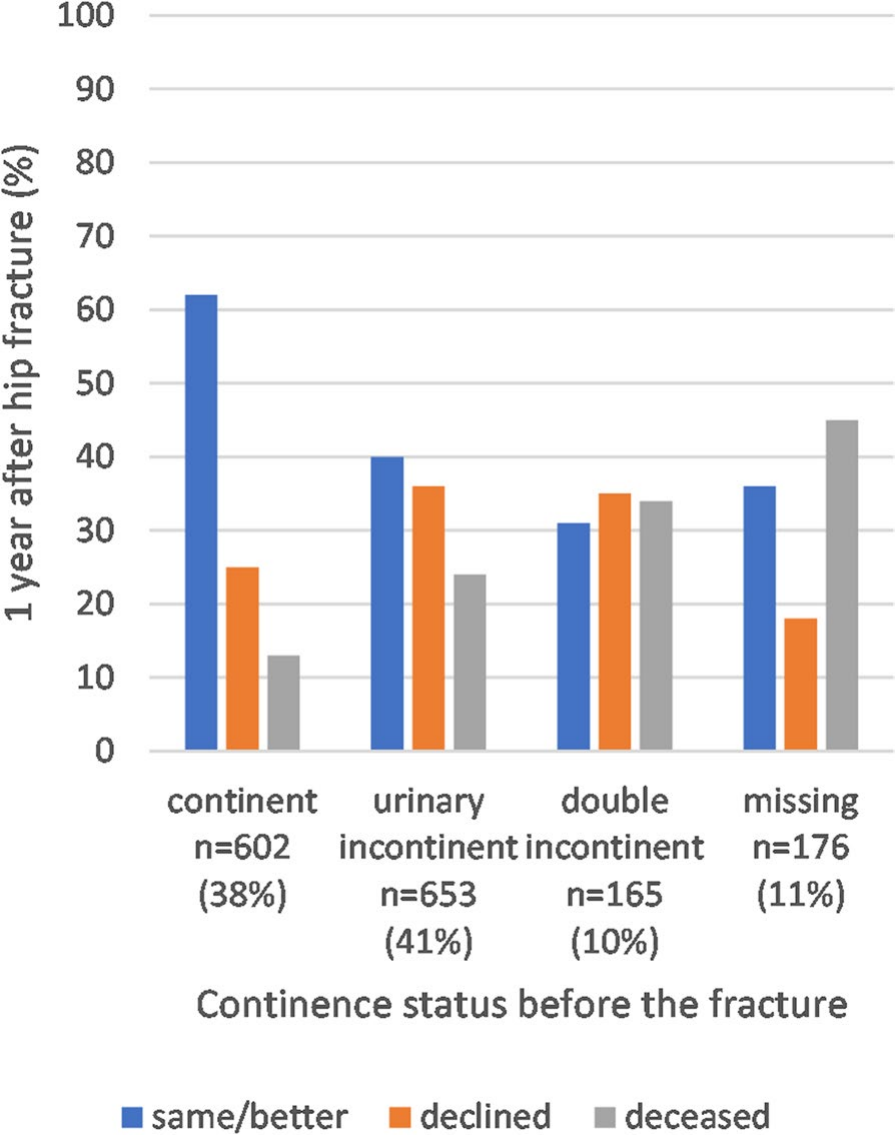
Changes in mobility after 1 year of follow-up according to pre-fracture continence status (*n* = 1596, 95% of the study population)

Changes in living arrangements and mobility according to the pre-fracture continence status are presented in Figs. 2 and 3. The continent patients had the highest rates of maintained mobility level and living arrangements. Patients with DI had the highest rate of need for more assisted living arrangements, and patients with UI or DI had the highest rate of declined mobility at 1-year post-fracture.

### Living arrangements outcome

Out of the 1,055 women, 551 (52%) lived independently in own home without services before the fracture and 504 (48%) lived in own home with the support of organized home care or in an assisted living accommodation, respectively. Out of the 551 formerly independently living women, 177 (32%) had required organized home care or moved to an assisted living accommodation, and 51 (9%) had institutionalized during the 1-year follow-up. Out of the 504 women with pre-existing services, 180 (36%) had institutionalized, and only 23 (2%) had required less services.

In the age-adjusted logistic regression analyses, pre-fracture UI (OR 1.33, 95% CI 1.01–1.76) and DI (OR 3.77, 95% CI 2.12–6.70) predicted the need for more assisted living arrangements at 1-year post-fracture, as well as all the other baseline variables except for the fracture type and number of regularly taken medications (Table 1). In the multivariable-adjusted analyses, pre-fracture DI (OR 2.40, 95% CI 1.22–4.75) continued to be associated with the need for more assisted living arrangements at 1-year post-fracture, but UI lost its predictive power. Age between 80 and 89 (OR 2.00, 95% CI 1.42–2.82), age over 90 (OR 4.30, 95% CI 2.65–6.98), diagnosis of cognitive disorder (OR 2.87, 95% CI 1.95–4.22), being able to ambulate outdoors with help (OR 3.61, 95% CI 2.47–5.27), being able to ambulate only indoors with help (OR 4.81, 95% CI 1.60–14.4), being unable to ambulate (OR 17.0, 95% CI 1.53–189) and late removal of UC (OR 1.67, 95% CI 1.22–2.30) also predicted the need for more assisted living arrangements at 1-year post-fracture. Conversely, having organized home care or living in an assisted living accommodation (OR 0.16, 95% CI 0.10–0.23) had a protective effect from need of further assistance at 1-year post-fracture (Table 1).

### Mobility outcome

Out of the 1,226 women, 714 (58%) could ambulate completely independently before the fracture, 454 (37%) required assistance outdoors, and 58 (5%) also indoors, respectively. Out of the 714 independent mobilizers, 209 (29%) required assistance when ambulating outdoors, 35 (5%) required assistance also indoors, and 15 (2%) had become unable to ambulate 1-year post-fracture. Out of the 454 women requiring assistance outdoors before the fracture, 31 (7%) had become independent mobilizers, 229 (50%) had maintained their mobility-level, 121 (27%) required assistance also indoors, and 73 (16%) had become unable to ambulate 1-year post-fracture. Out of the 58 women only able to ambulate indoors with assistance before the fracture, mobility-level improved in 9 (16%), was maintained in 25 (43%) and deteriorated to being unable to ambulate in 24 (41%) women 1-year post-fracture.

In the age-adjusted logistic regression analyses, pre-fracture UI (OR 2.11, 95% CI 1.63–2.75) and DI (OR 2.63, 95% CI 1.72–4.03) were associated with declining mobility at one-year, as well as all the other baseline variables except for pre-fracture mobility level (Table 2). The associations of UI (OR 1.88, 95% CI 1.41–2.51) and DI (OR 1.99, 95% CI 1.21–3.27) maintained their predictive power in the multivariable adjusted analysis, as well as age over 90 (OR 2.17, 95% CI 1.45–3.24), diagnosis of cognitive disorder (OR 2.00, 95% CI 1.45–2.77), requiring organized home care or living in an assisted living accommodation (OR 1.77, 95% CI 1.29–2.43), living in an institution (OR 2.56, 95% CI 1.58–4.15) and late removal of UC (OR 2.17, 95% CI 1.64–2.86). In addition, being able to ambulate outdoors with help (OR 0.40, 95% CI 0.29–0.57) and indoors only with help (OR 0.24, 95% CI 0.12–0.47) had a protective effect from further declining mobility-level (Table 2).

## Discussion

The principal finding of our study was that patients with pre-fracture DI had more than a two-fold risk of needing more assisted living arrangements compared to their continent counterparts 1-year post-fracture, whereas no such association was found with UI. Moreover, having UI or DI was associated with a two-fold risk of declining mobility-level 1-year post-fracture.

### Living arrangements outcome

Pre-fracture UI was not associated with the need for more assisted living arrangements in our study whereas DI was. Our findings are in accord with two previous studies carried out in general community-living older populations in which UI was associated with institutionalization in men but not in women [19, 21]. One study found that UI increased the risk of institutionalization independently of gender, but the risk was also greater in men than in women [20]. Authors of these studies have suggested that women tend to develop UI earlier in life and therefore it is not necessarily strongly associated with the onset or increasing disability in later years. This rationale is supported by a recent study, in which mild UI had a significant negative effect on quality of life of women, but increasing leakage resulted in only slight increase in this effect [32]. Another study by Finnish colleagues found that older women with FI reported lower health-related quality of life compared to their continent counterparts whereas no such association was found in women with UI [33]. In two studies comprising community-living older individuals, FI was associated with anxiety, depression, disability, and dependency [34, 35]. Incontinence also significantly burdens informal and formal caregivers resulting in both psychological and physical challenges [36]. Unsurprisingly, FI has been found to increase referrals to long-term care more markedly than UI [37].

Our own previous results support these findings given that older female hip fracture patients with DI had a significantly poorer physical and functional performance [24] and higher mortality rate compared to patients with UI only [25], thus also likely explaining the association with more need for assisted living arrangements during follow-up. FI is likely to constitute a more significant determinant for increasing disability and dependency compared to UI in these patients. It is also noteworthy that half of our study population had a clinical or emerging cognitive disorder [25, 38]. Given the high level of vulnerability in our study population, cognitive disorder may have an impact on the development of incontinence in our patients, and consequently in the need for more assisted living arrangements. However, studies examining incontinence as predictor of institutionalization in patients with cognitive disorders have had inconsistent results [39]. In all, to best of our knowledge, this is one of the first studies to clearly demonstrate DI as a predictor of institutionalization, albeit in a selected population of female hip fracture patients.

### Mobility outcome

Both pre-fracture UI and DI were predictors of decline in mobility in our study with nearly equal odds ratios. Previous research on incontinence as a predictor of decline in mobility is scarce. One study evaluated the connection between self-reported UI and decline in physical performance over a follow-up of two years in older community-living women. Short Physical Performance Battery (SPPB) was used. Women with UI performed worse and had a greater decline in SPPB compared to their continent counterparts. [40] In two reviews, a bidirectional relationship between UI and decline in physical function and mobility has been proposed: mobility limitations might amount to development of UI through weakened over-all skeletal muscle and pelvic floor function, and exacerbate existing UI, while UI may lead to voluntary reduction in the intensity and frequency of physical activity in fear of urine leakage [4, 41]. We found no studies directly examining the association of DI with decline in mobility. However, a cyclic relationship of declining physical function and FI has been suggested similar to that of UI [41]. We have now demonstrated these bidirectional relationships in our study population, since pre-fracture impaired mobility predicted incident UI and DI [25] and, pre-fracture UI and DI both predict decline in mobility-level 1-year post-fracture.

DI is also known to lead to greater decline in quality of life than UI and FI in isolation [14–16], presumably resulting in social isolation and decline in physical activity. It may thus be hypothesized that DI might lead to an even greater decline in mobility than UI and FI in isolation. It is therefore interesting that DI didn’t have significantly stronger odds ratio than UI in our study. Reduced statistical power due to the limited sample of patients with DI might partly explain this.

Since mobility seems to have a role in the pathophysiology of incontinence in older women [4, 41], and physical activity has been noted to protect from incident UI [26] and FI [42], interventions aimed at improving physical performance could potentially prevent new-onset incontinence and improve existing incontinence in older women. Some benefit of exercise programs has been demonstrated in small clinical trials in the management of UI [43, 44]. Indeed, early rehabilitation aimed at regaining pre-fracture mobility level likely serves both the over-all recovery and prevention or management of incontinence in older women after a hip fracture.

Interestingly, in our earlier study carried out with a shorter follow-up of 4 months after the hip fracture, rates of declined mobility (39%) and need for more supported living arrangements (38%) were nearly the same [45] as in present study, which suggests that the deterioration of mobility and the need for more assisted living arrangements occur in the early stages of recovery. This further highlights the need for focusing resources on the rehabilitation in the first months following a hip fracture. According to a recent Cochrane review, a multidisciplinary rehabilitation approach after a hip fracture may decrease the likelihood of poorer mobility and institutionalization at 6–12 months’ follow-up after a hip fracture in older people [46].

Unsurprisingly, the significant association of other predictor variables, which were used as covariates in the present study, with both examined outcomes were in accord with our previous observations [27].

In addition, a significant association between poor nutritional status as measured by the MNA-SF and both decline in mobility and the need for more supported living arrangements was observed, but only in the age-adjusted analyses. We have previously demonstrated malnutrition to be extremely common among older female hip fracture patients and associated with DI 6 months post-fracture [24]. We have also established malnutrition as an independent predictor of increased mortality and a marker of disability in these patients [25]. As in our previous studies, the fact that our multivariable model was adjusted for living arrangements and mobility level likely explains why malnutrition lost its predictive value in the final results.

### Strengths and limitations

According to best of our understanding, this was one of the first studies to establish DI as a predictor of institutionalization and decline in mobility, and one of the few existing studies to examine these associations for UI in female hip fracture patients. The strength of our study was its real-world prospective and population-based design and systematic data collection. Importantly, our results are representative of female hip fracture patients given that both patients living in LTCFs and having cognitive disorders were included in the study.

We acknowledge several limitations which should be considered when interpreting our results. First, incontinence symptoms were evaluated only with simple questions instead of validated questionnaires, and frequency, UI type, severity or time frame of the symptoms were not included in the data collection. This was done in order to reach as high response rates as possible given the cognitively impaired and disabled patient population. Moreover, the patients’ representatives were used as proxies in cases where the patients were unable to answer the questions themselves. According to an earlier report, a relatively good concordance was observed between index and proxy answers for questions on both UI and FI among older patients with a hip fracture [47]. Second, given the small number of observations in the FI only group, this was excluded from the analyses. Third, comorbidities were not recorded in detail, and possible pre-existing urogynecological disorders were not known. Only ASA score represents the general health of the patient. Fourth, due to the relatively long period of data collection, there could be secular changes in the patient population and in the care they have received, which may have affected the results. Fifth, considering the relatively small sample sizes of the DI groups, some caution is due when interpreting our results. Sixth, our study concerned only women with hip fracture. This was due to the fact that both hip fractures and continence problems are known to be notably more common in women than in men. Examining the effect of incontinence on hip fracture outcomes in male patients is also warranted in future studies. Finally, there is a possibility of both selection and survivor bias, as every fifth patient died during the follow-up and rates of incontinence and disability are expected to be high in these patients. Those in poorest health among the survivors were also most likely to drop out, which might result in lower rates of institutionalized and immobilized patents. It is also worth noting that due to differences in the structure and availability of home-based and long-term care services, the results on changes in living arrangements observed in the present study may not be directly generalizable to other societies.

## Conclusions

Pre-fracture UI and DI predict decline in mobility and DI the need for more assisted living arrangements 1-year post-fracture in older women. These findings highlight the need to focus on regaining pre-fracture mobility level and managing incontinence during the early rehabilitation process after a hip fracture.

## Data Availability

The datasets generated and analysed during the current study are not publicly available due to limitations of ethical approval involving the patient data and anonymity but are available from the corresponding author on reasonable request.

## Abbreviations

UI: Urinary incontinence
FI: Fecal incontinence
DI: Double incontinence
UC: Urinary catheter
ASA: The preoperative American Society of Anesthesiologists
MNA-SF: Mini Nutritional Assessment Short Form
